# Acoustic spectral feedback in vocal learning: a systematic review of psychological mechanisms and skill outcomes

**DOI:** 10.3389/fpsyg.2026.1920074

**Published:** 2026-08-20

**Authors:** Yuze Zhang

**Affiliations:** School of Music, Harbin Normal University, Harbin, China

**Keywords:** acoustic spectral feedback, guidance hypothesis, metacognition, motor learning, self-efficacy, self-regulated learning, vocal learning

## Abstract

**Background:**

Vocal skill acquisition is inherently “invisible”: learners cannot directly observe the movements of their vocal organs, so traditional instruction depends heavily on the teacher’s auditory judgment and metaphorical language. This leaves learners ill-equipped to detect errors and self-correct during independent practice. Spectral and acoustic visualization technologies—pitch-trajectory displays, spectrograms, formant analysis, and physiological biofeedback—can externalize this hidden process, yet systematic synthesis at the level of psychological mechanisms remains scarce.

**Aim:**

Using psychological mechanisms as its organizing framework, this review synthesizes the role of acoustic spectral feedback in vocal learning and seeks to bridge motor learning theory and self-regulated learning theory, which have not previously been integrated in this context.

**Methods:**

Following PRISMA 2020, we systematically searched WoS, Scopus, PsycINFO, ERIC, PubMed, and CNKI (2005–2025). Using thematic narrative synthesis, we classified the 36 included studies by psychological mechanism rather than by tool type; only 7 reported a formal mediation test.

**Results:**

The evidence revealed five mechanism pathways. Spectral feedback is associated with self-efficacy gains attributed to accumulated mastery experience via knowledge of results (relatively strong). It promotes metacognitive monitoring by externalizing implicit mechanisms (strong for measurement, but the metacognition→skill test was null). It supports self-regulated learning through the plan–monitor–reflect cycle (moderate). Its effect on motivation and emotional engagement remains unverified (limited). Real-time concurrent feedback entails a cognitive-load versus guidance-dependency trade-off (moderate). Critically, 29 of 36 studies did not formally test the feedback→mechanism→skill pathway—the field’s most prominent methodological gap.

**Conclusion:**

A preliminary integrated pathway framework of acoustic spectral feedback in vocal learning is advanced as an interpretive proposition derived from the synthesized evidence rather than as an empirically verified mechanism, with evidence gaps and testable hypotheses annotated so as to scaffold future empirical research.

**Systematic review registration:**

Open Science Framework, https://osf.io/qgwxk, identifier qgwxk.

## 1 Introduction

For a long period of history, vocal pedagogy has relied on a distinctive mode of knowledge transmission. The teacher uses a trained ear to detect deviations in the student’s voice, and then guides the student to adjust their vocal strategy through auditory description and metaphorical language, such as “place the voice in the head resonator” or “imagine the breath supporting the tone” (Morales-Villar et al., 2023; Welch et al., 2005). This mode may be effective in one-to-one master–apprentice settings. Its fundamental limitation, however, is that the real-time movements of the larynx, the vocal tract, and the diaphragm are entirely invisible to the learner. Learners can neither directly observe their own physiological state at the moment of phonation nor rely on an objective acoustic reference to verify the gap between self-perception and actual output (McPherson et al., 2022; Bernardini et al., 2022). This “hidden” quality makes the acquisition of vocal skill inherently subject to a challenge during independent practice. When the teacher is absent, learners often cannot judge whether their voice is accurate, and the efficiency of practice is therefore greatly reduced.

The emergence of spectral and acoustic visualization technologies offers an operable means of externalizing the difficulty described above. Pitch-trajectory displays can present pitch deviations at the moment of phonation on screen with pixel-level precision. Spectrograms and formant analysis can transform vocal-tract resonance characteristics—a dimension of vocal quality that is crucial to bel canto yet almost impossible to verbalize—into observable spectral patterns. Physiological biofeedback can externalize underlying physiological indices, such as breath pressure and vocal-fold vibration, into real-time visual signals (Bernardini et al., 2022; Paney and Tharp, 2021; Moss, 2021). In terms of timing, these tools can be further distinguished into concurrent feedback, which is presented in real time during phonation, and terminal feedback, which is presented after phonation ends. The two differ in essential ways at the cognitive level, and this distinction is crucial for understanding how feedback influences the learning process (García-García and Tejada, 2021).

Existing research on vocal visualization feedback shows a clear technology-oriented bias in its research focus. Related reviews and empirical studies have concentrated mostly on describing tool functions, evaluating software performance, and testing direct skill effects, whereas systematic verification of psychological mechanisms remains relatively scarce (Blackwell et al., 2023; Chen and Lin, 2026). This judgment is not an isolated impression but has direct support in the literature. A recent study of AI-assisted music practice applications explicitly noted that most research focuses on the development of technical functions and lacks rigorous verification of self-efficacy and performance outcomes (Ou et al., 2025). Lubert and Gröpel (2025), in their systematic review of psychological interventions for music performance, likewise revealed a similar structural gap: intervention research tends to focus on effects rather than on activating mechanisms. This means that our current understanding of why spectral feedback works is far less developed than our understanding of whether it works.

From the perspective of theoretical resources, two complementary frameworks both hold application potential in this field, yet neither has been systematically integrated within the context of vocal spectral feedback. Motor learning theory—especially the distinction between knowledge of results (KR) and knowledge of performance (KP), the guidance hypothesis, and Schmidt’s schema theory—provides a cognitive–motor account of why visually assisted practice can promote skill automaticity (Sweller, 2020; Jimenez-Diaz et al., 2021). At the same time, educational psychology frameworks—including Zimmerman’s three-phase cycle of self-regulated learning, Flavell’s theory of metacognition, and Bandura’s theory of self-efficacy—explain, at the cognitive–motivational level, how a learner’s internal psychological processes mediate the effect of external feedback on skill outcomes (López-Íñiguez and McPherson, 2020; Peistaraite and Clark, 2020; Hatfield et al., 2017). The two frameworks cover different levels of analysis. How to integrate them within the specific context of vocal spectral feedback is a question that no study has yet systematically attempted to answer.

The analysis above converges on one unresolved tension that organizes the present review, namely the widening distance between what the field has demonstrated and what it has explained, since a substantial body of work now indicates that spectral and acoustic visualization can improve measurable vocal performance, whereas the psychological processes assumed to carry that improvement are for the most part asserted on theoretical grounds rather than established through pathway testing. Four questions are derived from this tension and are answered directly in Section 4.1. RQ1 concerns the affordances that acoustic spectral feedback provides in vocal learning and the manner in which these affordances can be classified by the nature and the timing of the feedback. RQ2 concerns the psychological mechanisms that are proposed to mediate the relationship between spectral feedback and vocal skill outcomes, together with the density of the direct measurement evidence available for each of them. RQ3 concerns the extent to which existing research supports these mechanism-to-skill links through formal mediation testing rather than through verbal inference. RQ4 concerns the design gaps that follow from the resulting evidence landscape and the configuration of future studies capable of closing them. In terms of research contribution, this review synthesizes research on vocal spectral feedback with psychological mechanisms as its explicit analytical axis, an organizing choice that the reviews closest to it in orientation have not adopted. By integrating the two theoretical frameworks of motor learning and self-regulated learning, it advances a preliminary “affordance→psychological mechanism→skill outcome” framework (Wang et al., 2025), which is offered as an interpretive proposition derived from the synthesized evidence rather than as an empirically verified mechanism, and whose causal pathways await verification by future studies designed with formal mediation tests.

## 2 Methods

### 2.1 Study design and registration

This study adopted a narrative/thematic synthesis systematic review design rather than a meta-analysis. There were three reasons for this methodological choice. First, the included studies were highly heterogeneous in design type, sample characteristics, and measurement tools. Second, the vast majority of studies did not report effect sizes that could be directly extracted. Third, very few studies formally tested the feedback→mechanism→skill mediation pathway, which made it impossible to meet the statistical prerequisites for a meta-analysis. The review materials were registered on the Open Science Framework (OSF; https://osf.io/qgwxk; identifier: qgwxk). The OSF record documents the review protocol, search strategy, screening, and decisions, coding framework, and synthesis procedures for transparency and reproducibility, in line with transparent reporting principles for systematic reviews (Page et al., 2021).

### 2.2 Search strategy

Following the PCC (Population–Concept–Context) framework, we constructed a three-concept search string and conducted a systematic search across five English-language databases—WoS Core Collection, Scopus, APA PsycINFO, ERIC, and PubMed—as well as the Chinese-language database CNKI. The time window was set from 2005 to August 2025. We explicitly included two foundational empirical studies published in 2005 and 2006 (Welch et al., 2005; Hoppe et al., 2006). The reason was that these two studies established the core experimental paradigm of visual-feedback-assisted vocal training and therefore have an irreplaceable anchoring value for understanding the theoretical lineage of subsequent mechanism research (Page et al., 2021).

The core search string was as follows:

(singing OR “vocal learning” OR “voice training” OR “singing pedagogy”) AND (“acoustic feedback” OR “spectral feedback” OR “visual feedback” OR “real-time feedback” OR “pitch display” OR “spectrogram” OR “formant” OR “biofeedback”) AND (“self-efficacy” OR “metacognition” OR “self-regulated learning” OR “cognitive load” OR “motivation” OR “psychological mechanism” OR “skill outcome” OR “pitch accuracy”)

The initial search yielded 2,010 records. With the addition of 64 records from the supplementary CNKI search, the total was 2,074 records, and 1,820 records remained after deduplication (Page et al., 2021). In addition, to include peripheral studies that tested the same psychological mechanisms in the context of instrumental pitch/intonation visualization feedback, we performed a further targeted search built around feedback-mechanism terms and with relaxed instrument restrictions. Such studies were retained only when the psychological mechanism they tested was transferable to the vocal context.

### 2.3 Inclusion and exclusion criteria

*Inclusion criteria*: The study participants were primarily vocal learners (singing learners and music-major students receiving vocal training). For high-quality studies that tested the same psychological mechanisms (self-efficacy, metacognition, SRL, and the like) in the context of instrumental (e.g., violin/viola) pitch–intonation visualization feedback, we included them on a limited basis as cross-domain peripheral evidence at mechanism nodes where vocal evidence was scarce, and we clearly noted their non-vocal nature in Table 1 and in the main text. The intervention or exposure had to involve the use of an acoustic/spectral visualization tool. The reported outcomes had to cover a psychological variable (any one of self-efficacy, metacognition, SRL, motivation, or cognitive load) or a quantifiable vocal skill indicator (pitch accuracy, resonance/timbre, breath management, or overall singing rating). The publication form had to be a peer-reviewed journal article.

**Table 1:** Summary of the characteristics of included studies (selected representative studies).

Author/year	Sample (*N*/type)	Feedback type	Timing	KR/KP	Psychological outcome	Skill outcome	Mediation test
Welch et al. (2005)	12/vocal students	Pitch trajectory	Concurrent	KR	Self-monitoring (qualitative)	Pitch accuracy	No (indirect inference)
Paney and Tharp (2021)	48/adults	Pitch trajectory	Concurrent	KR	Self-efficacy (scale)	Pitch accuracy (SSAP)	No (descriptive)
Berglin et al. (2022)	60/adults	Pitch trajectory	Concurrent	KR	Not measured	Pitch accuracy (SSAP)	No
Moss (2021)	Case/professional singers	Spectrogram, formant	Concurrent	KP	Metacognition (qualitative)	Resonance / timbre	No (descriptive)
García-García and Tejada (2021)	32/music majors	Pitch trajectory	Concurrent / Terminal	KR/KP	Self-monitoring (qualitative)	Pitch accuracy	No (descriptive)
Li et al. (2025)	80/pre-service teachers	Audio waveform comparison	Terminal	KR	Metacognition (scale)	Overall singing (APRIOSV)	Yes (LMM)
Ou et al. (2025)	40/violin majors	Pitch-tracking app	Concurrent / Terminal	KR/KP	Self-efficacy (MLSE/MPSE)	Performance score	Yes (LMM)
López-Calatayud and Tejada (2024)	4/beginner string students	Intonation assessment software	Concurrent	KR	Self-regulation (qualitative)	Intonation	No (qualitative)

KR = knowledge of results; KP = knowledge of performance; SSAP = Seattle singing accuracy protocol; APRIOSV = auditory-perceptual rating instrument for the operatic singing voice; MLSE = music learning self-efficacy; MPSE = music performance self-efficacy; LMM = linear mixed-effects model. This table presents representative studies only.

*Exclusion criteria*: We excluded purely engineering or acoustic signal-processing studies that did not report a learning outcome; studies focused on the clinical intervention of voice disorders (i.e., non-instructional settings); literature not in English or Chinese; and conference abstracts, dissertations, and unpublished manuscripts (Peters et al., 2020).

### 2.4 Screening procedure

**Figure 1:**
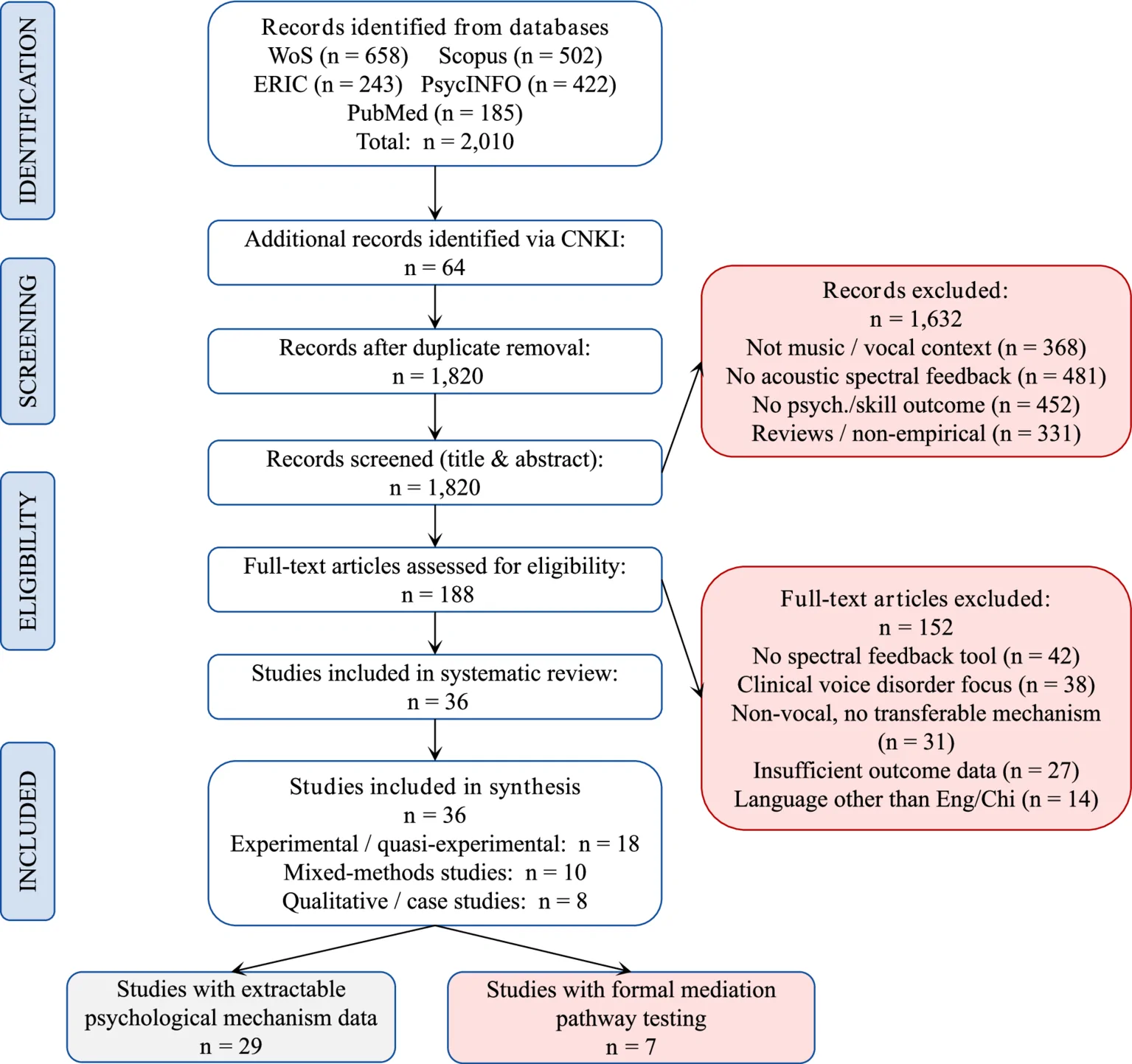
PRISMA flow diagram. WoS = web of science; CNKI = China national knowledge infrastructure. Search period: 2005–2025. Foundational studies (Hoppe et al., 2006; Welch et al., 2005) retained with documented justification.

Screening was conducted in two stages. In the title/abstract screening stage, the author excluded 1,632 records and retained 188 articles for full-text assessment. In the full-text assessment stage, 152 articles were excluded, and 36 articles were finally included. As this review was conducted by a single author, to reduce selection error the entire screening process was performed twice with a two-week interval, and any inconsistencies between the two passes were resolved by re-examining the full text against the inclusion criteria. Figure 1 presents the complete screening process.

### 2.5 Data extraction

Data were extracted using a predefined structured charting form. The extracted fields included the study sample (*N*/type), the feedback type (spectrogram, pitch trajectory, formant analysis, biofeedback), the feedback timing (concurrent/terminal), the feedback nature (KR/KP), the psychological outcome (measurement tool and direction of result), the skill outcome (measurement tool and direction of result), and whether a formal mediation pathway test was reported (yes/no/indirect inference). Data extraction was performed by the author using the predefined charting form. To minimize extraction error, all extracted entries were independently re-checked against the original articles in a second pass, and any discrepancies were corrected by returning to the source text (Peters et al., 2020). Table 1 presents a summary of the core characteristics of the included studies.

### 2.6 Quality appraisal

**Figure 2:**
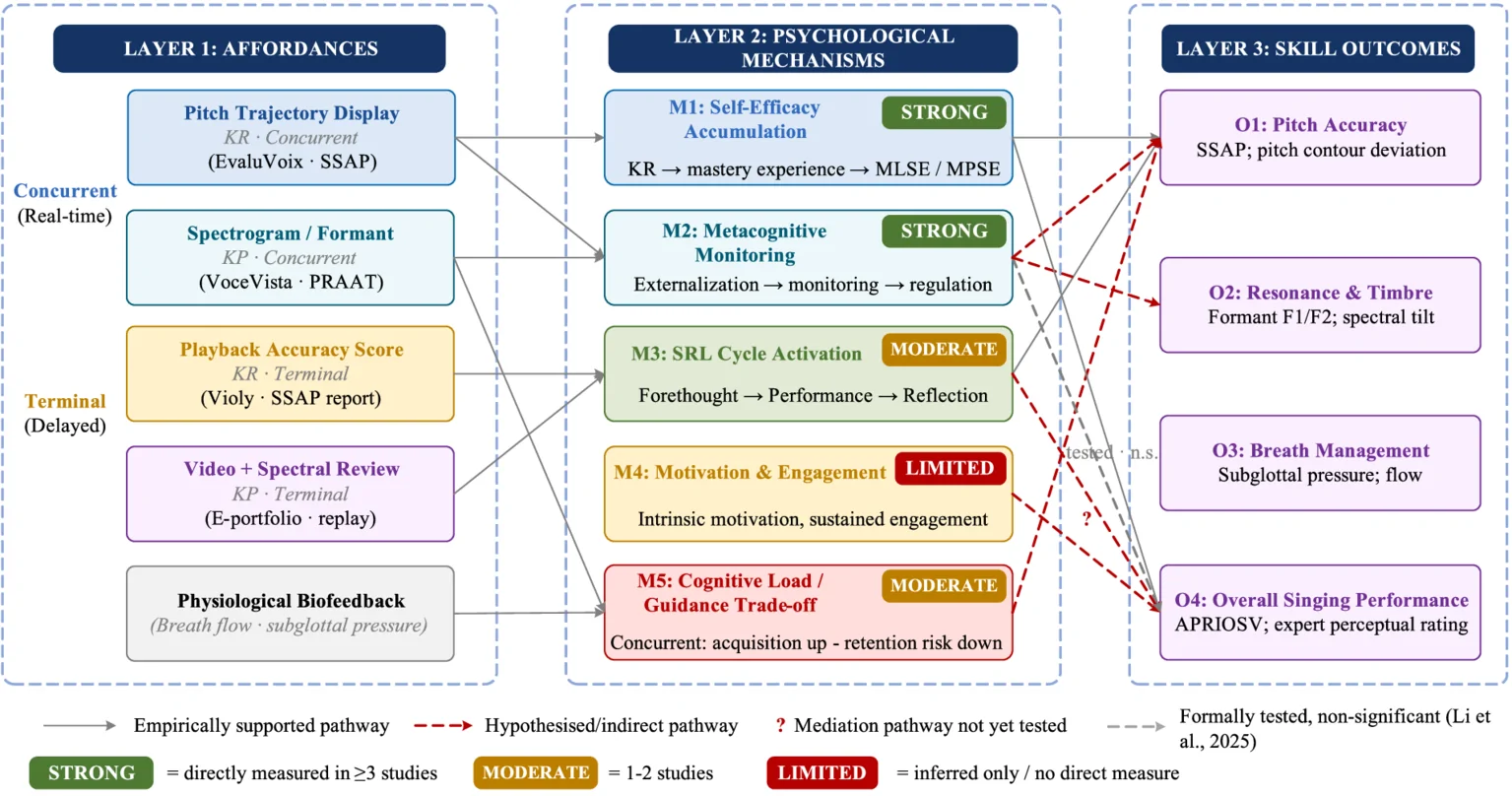
Preliminary integrated conceptual framework of acoustic spectral feedback, psychological mechanisms, and vocal skill outcomes. Solid arrows = links for which at least one included study reported a formal mediation test returning a significant and positive estimate. Dashed arrows = links resting on indirect inference or on evidence insufficient for a pathway claim. “?” = links for which no included study reported a formal mediation test. This framework is preliminary and intended to guide future empirical testing, not to assert causal claims. The STRONG/MODERATE/LIMITED badges denote only the density of studies directly measuring each mechanism, not the strength of any “mechanism → skill” causal path; path-testing status is reported separately in Table 2.

We used the Mixed Methods Appraisal Tool (MMAT) to assess the methodological quality of the included studies. The appraisal dimensions included the appropriateness of the study design, the reporting of the reliability and validity of measurement tools, the level of control over sources of bias, and the degree of fit between conclusions and data. The quality appraisal results were not used as exclusion criteria. The MMAT ratings were used in the Discussion to explain differences in results across studies. By contrast, the evidence-strength badges in Figure 2 were defined according to the number of studies that directly measured each mechanism (≥3 studies = STRONG, 1–2 studies = MODERATE, indirect inference only = LIMITED). The two metrics use different criteria, and each has been labeled separately.

### 2.7 Synthesis method

The unit of synthesis was the psychological mechanism rather than the tool type. This choice constitutes the fundamental difference, in organizing logic, between this review and previous tool-oriented reviews (Blackwell et al., 2023; Wang et al., 2025). Under each mechanism node, we first present the direct evidence, then annotate the indirect inferences, and finally identify the pathway-testing gap clearly, treating the “evidence gap” itself as a valid component of the synthesized conclusion.

### 2.8 Analytic trustworthiness

Because the entire review was designed, screened, charted, and synthesized by a single author, no collaborative coding, independent inter-coder checking, or external audit of the analytic decisions was carried out, and the credibility of the synthesis therefore rests on procedures that constrain individual interpretation rather than on agreement between coders. The coding frame was fixed before synthesis began and was derived deductively from five theoretical constructs, namely self-efficacy, metacognition, self-regulated learning, motivation together with emotional engagement, and cognitive load together with guidance dependency, so that the assignment of a study to a mechanism node followed the constructs that the study itself measured rather than an impression formed during reading. These constructs are documented in the OSF record. Screening, data charting, and mechanism assignment were each performed twice with a two-week interval, and every inconsistency between the two passes was resolved by returning to the full text and adjudicating against the written criteria rather than against the earlier decision. A deliberate search for disconfirming evidence accompanied each mechanism node, which is the reason that the non-significant metacognition→performance result reported by Li et al. (2025) and the retention-test convergence reported by Paney and Tharp (2021) are foregrounded in the synthesis rather than subordinated to the supportive findings. The review protocol, the search log, the charting form, and the record of screening decisions are retained in the OSF project record so that the analytic trail can be inspected and reconstructed independently. These measures reduce, without eliminating, the risk of single-analyst bias, and the residual risk is acknowledged as a limitation in Section 4.6.

## 3 Results

### 3.1 Overview of included studies

A total of 36 studies were finally included, with a combined sample size of approximately 2,850 participants (*N* range: 1–334; median *N* = 48). The distribution by year of publication showed 6 studies in 2005–2015, 9 studies in 2016–2020, and 21 studies in 2021–2025. Studies from the most recent 5 years account for about 58% of the total, which reflects the rapid development of this field in recent years. In terms of study design, experimental or quasi-experimental designs predominated (18 studies, 50%), followed by mixed-methods studies (10 studies, 28%) and qualitative/case studies (8 studies, 22%). The geographical distribution was concentrated in Europe (the United Kingdom, Spain, Norway) and East Asia (mainland China, Taiwan, China). Studies from Asia accounted for about 39%, of which 14 were included through the supplementary CNKI search. This composition suggests that recent Asian research output in this field deserves attention (Wang et al., 2025).

Among the 36 included studies, 22 were core studies that simultaneously satisfied the three elements of “spectral feedback + psychological mechanism measurement + vocal context.” The remaining 14 were either instrumental peripheral evidence lacking one of these elements or skill-only studies. Across all 36 studies, 29 (81%) measured and reported extractable psychological outcome data, whereas only 7 (19%) formally and statistically tested the feedback→mechanism→skill mediation pathway (for example, through structural equation modeling or multilevel linear modeling). Because several of these 7 studies were instrumental peripheral evidence, the proportion of pathway testing reported above should be interpreted against the background of the three-element core subset (22 studies). This distribution of proportions is in itself one of the core findings of this review, as it points directly to the methodological gap in the current field. Table 1 presents a complete summary of the characteristics of the included studies, covering feedback type, the dimensions of psychological mechanisms involved, skill outcome measurement, and mediation-testing status.

### 3.2 Classification of the affordances of spectral/acoustic feedback

**Figure 3:**
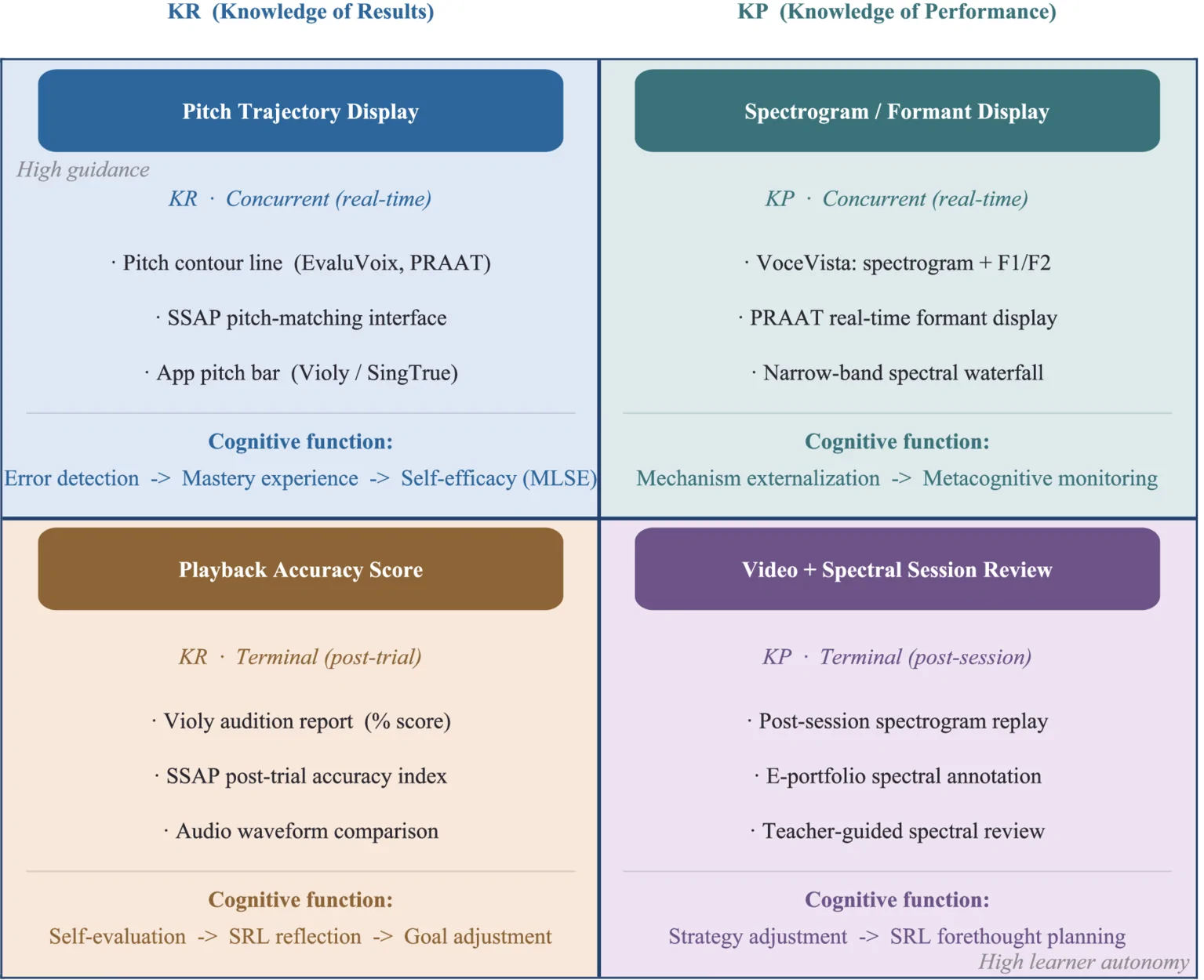
Affordance classification matrix of acoustic spectral feedback in vocal learning feedback nature. KR = knowledge of results (outcome-level accuracy information, e.g., pitch deviation score). KP = knowledge of performance (process-level acoustic/biomechanical information, e.g., formant pattern). SSAP = Seattle singing accuracy protocol; MLSE = music learning self-efficacy; SRL = self-regulated learning. Diagonal markers indicate gradient from externally guided (top-left) to learner-autonomous (bottom-right) practice. Tool examples are representative, not exhaustive.

The spectral/acoustic feedback tools involved in the included studies can be organized into a 2 × 2 classification matrix along two dimensions: the nature of the feedback (KR vs. KP) and the timing of the feedback (concurrent vs. terminal) (as shown in Figure 3). Pitch-trajectory displays (such as EvaluVoix and the SSAP companion interface) are a form of real-time KR feedback. They can provide a quantified result for pitch deviation at the moment of phonation, and their cognitive function is mainly expressed in error detection and immediate correction. Spectrograms and real-time formant analysis (such as VoceVista and the PRAAT real-time display) are a form of real-time KP feedback. They externalize the phonatory mechanism itself—vocal-tract shape and resonance configuration—into observable spectral features, and their cognitive function lies in moving the learner from outcome monitoring toward an understanding of the mechanism (Bernardini et al., 2022; Oppici et al., 2021). The typical form of terminal KR feedback is an accuracy score presented after a complete singing segment (such as the audition report in Violy). Its cognitive function lies mainly in supporting self-assessment and the reflection cycle. Terminal KP feedback takes the form of a teacher-guided review combined with a spectral recording after the lesson, or a spectral annotation in an electronic practice portfolio. It mainly supports strategy adjustment and goal setting for the next practice session (García-García and Tejada, 2021; Paney and Tharp, 2021).

It is worth noting that this classification is not a simple enumeration of technical features. Rather, it corresponds to different cognitive mechanisms. Real-time KR mainly activates the pathway of error detection→mastery experience→self-efficacy. Real-time KP mainly supports the pathway of mechanism externalization→metacognitive monitoring. Terminal feedback (whether KR or KP) acts more directly on the self-reflection phase of the Zimmerman SRL cycle.

Figure 3 reveals a structural asymmetry that has not been explicitly discussed in previous tool-oriented reviews. Among the included studies, the experimental evidence for the KR-concurrent quadrant (pitch-accuracy training) is the richest, whereas the controlled experimental evidence for the KP-concurrent quadrant (real-time formant/spectral display) and the KP-terminal quadrant (spectral recording review) is clearly thin. This uneven distribution reflects researchers’ tool preferences. It also means that, for resonance-regulation training—the most discipline-specific aspect of vocal learning—the existing evidence base remains rather limited (Moss, 2021; Oppici et al., 2021).

### 3.3 Mechanism one: self-efficacy accumulation

Self-efficacy—an individual’s judgment of their confidence in completing a specific task—is a core psychological variable that predicts persistence in music learning and the quality of strategy use (Ritchie and Williamon, 2011; Zelenak, 2024). In the context of spectral feedback, KR-type tools create reproducible mastery experiences for learners by providing objective, quantified performance scores. Mastery experience is the strongest source of efficacy beliefs in Bandura’s theory of self-efficacy (Ou et al., 2025). Compared with purely subjective perception, the objectivity of spectral tools helps to offset the efficacy bias that arises from excessive self-criticism or blind overconfidence, and thereby helps learners form a more calibrated judgment of their own ability (Berglin et al., 2022; Paney and Tharp, 2021).

Ritchie and Williamon's (2011) two-dimensional distinction in musical self-efficacy—learning self-efficacy (MLSE, confidence in the learning and preparation process) and performance self-efficacy (MPSE, confidence in actual singing/playing performance)—provides a more refined analytical framework in the context of spectral feedback. The quasi-experimental study by Ou et al. (2025) showed that an AI-assisted practice application had a greater enhancing effect on MPSE (effect size d = 0.58) than a protective effect on MLSE. This dissociation pattern suggests that the influence of spectral feedback tools on performance confidence may operate through a mechanism different from that for learning confidence. The former relies more on positive feedback from objective performance data, whereas the latter involves more the management of perceived practice difficulty. It should be noted, however, that the study cited above used a violin rather than a vocal setting, and the transferability of its conclusions to the vocal domain awaits dedicated research for verification (Zelenak, 2024).

### 3.4 Mechanism two: metacognition and self-monitoring

Metacognition—the monitoring and regulation of one’s own cognitive processes—is a psychological construct that is often invoked yet rarely rigorously measured in music skill learning (Concina, 2019; Wang et al., 2025). In vocal learning, metacognitive monitoring faces a fundamental obstacle: learners cannot “see” their own voice at the moment of phonation, and their self-perception often shows a systematic deviation from the actual acoustic output (Li et al., 2025; Li et al., 2023). By externalizing the implicit phonatory mechanism into observable visual signals, spectral visualization tools provide a “third-person perspective” for metacognitive monitoring. Learners can treat the spectral pattern on the screen as an objective record of their own phonatory behavior, and then carry out comparison, judgment, and strategy adjustment (Bernardini et al., 2022).

The experimental study by Li et al. (2025) provided direct evidence that an audio-comparison tool promotes metacognitive development. After a six-week intervention, the experimental group scored significantly higher on the metacognition scale than the control group [*F*(1, 78) = 5.10, *p* = 0.03, *η*^2^*p* = 0.06]. A qualitative thematic analysis further revealed that, by repeatedly listening to their own recordings, students discovered problems they had never noticed before. However, the same study reported a noteworthy null result: the improvement in metacognition did not significantly predict an improvement in singing performance (*t* = 0.23, *p* = 0.82). This suggests that the influence of metacognition on vocal skill may need to be realized through mediating variables (such as the quality of strategy use or the allocation of practice time) rather than through a direct effect (Li et al., 2025; Wang et al., 2025). This finding carries an important cautionary message for establishing the complete spectral feedback→metacognition→skill pathway: externalization tools can enhance monitoring awareness, but turning monitoring into skill improvement still requires the intervention of additional regulatory mechanisms (Concina, 2019).

### 3.5 Mechanism three: the self-regulated learning cycle

Zimmerman’s three-phase cycle of self-regulated learning (SRL)—forethought, performance, and self-reflection—has been shown by several studies to align closely with the high-quality practice behavior of professional musicians (López-Íñiguez and McPherson, 2020; Hatfield et al., 2017; Dos Santos Silva and Marinho, 2025). Spectral feedback tools support all three phases, but the mechanisms of support differ. In the forethought phase, historical performance data (such as the accumulated records in the Violy audition report) provide a basis for goal setting and strategy planning that is grounded in objective data rather than subjective perception (Ou et al., 2025). In the performance phase, a real-time pitch trajectory or spectral display supports self-monitoring during practice, which enables learners to identify deviations at the moment a problem occurs rather than afterward (Bernardini et al., 2022; Paney and Tharp, 2021). In the self-reflection phase, a terminal spectral recording or audio playback provides objective material for evaluating the completion of the current goal and for planning the next practice session (López-Calatayud and Tejada, 2024; Wan et al., 2023).

Dos Santos Silva et al.'s (2023) analysis of a social-media music practice challenge (#100daysofpractice) showed that music learners who actively recorded and shared their practice process displayed a more complete SRL cycle. This indicates that externalization tools (including recording and visualization feedback) have a structural function in maintaining the coherence of the SRL cycle. However, the existing evidence is mostly correlational research or qualitative description. To date, no study has used an experimental design to directly test, in the context of vocal spectral feedback, the complete chain of “tool use→improved SRL quality→skill outcome,” and this remains a gap (Dos Santos Silva and Marinho, 2025; Wang et al., 2025).

### 3.6 Mechanism four: motivation and emotional engagement

Among the five psychological mechanisms, motivation and emotional engagement have the weakest evidence. Among the 36 included studies, no more than 5 directly measured emotional or motivational outcomes with standardized scales, and these studies differed considerably in their measurement time points and in how results were reported, which made cross-study comparative synthesis difficult (Peistaraite and Clark, 2020; Song et al., 2024; McPherson et al., 2022). The existing evidence comes mainly from two types of indirect source. The first is the motivational feeling that learners spontaneously mention in qualitative interviews or reflective journals (for example, “being able to see my own progress makes me want to keep practicing”). The second is the use of practice adherence rate or practice time as a behavioral proxy indicator of motivation (Ou et al., 2025).

Peistaraite and Clark (2020) found that emotion reappraisal can reduce the interference of negative emotions in the SRL process and free up cognitive resources for strategic practice. This suggests that emotion regulation may act as an intermediate variable through which spectral feedback influences SRL quality. However, this hypothesized pathway currently relies entirely on theoretical deduction, and no direct test has been conducted in the context of vocal spectral feedback. Incorporating emotional/motivational outcomes into the measurement system of future research is one of the priorities for methodological development in this field (McPherson et al., 2022; Song et al., 2024).

### 3.7 Mechanism five: the trade-off between cognitive load and guidance dependency

The guidance hypothesis is one of the most theoretically influential frameworks for explaining the effects of augmented feedback in the field of motor learning. Concurrent feedback raises the performance level during the acquisition phase because it provides continuous error-correction information during training. For exactly the same reason, however, learners tend to substitute the feedback signal for the construction of an internal sensorimotor model. Once the feedback is removed, performance often shows a marked decline (McKay et al., 2022; Drews et al., 2024). This two-phase pattern of “improvement during training, decline after feedback removal” has received partial empirical support in research on vocal visualization feedback. Paney and Tharp (2021) found that, although a group given 10 weeks of concurrent visual feedback performed better during training, its retention-test scores after feedback removal converged with those of the control group, which suggests the potential risk of guidance dependency. The study by Berglin et al. (2022) showed that visual feedback training produced a clearer long-term acquisition benefit for learners with low initial pitch accuracy, which implies that the degree of feedback dependency may be moderated by the learner’s initial ability level (García-García and Tejada, 2021).

**Figure 4:**
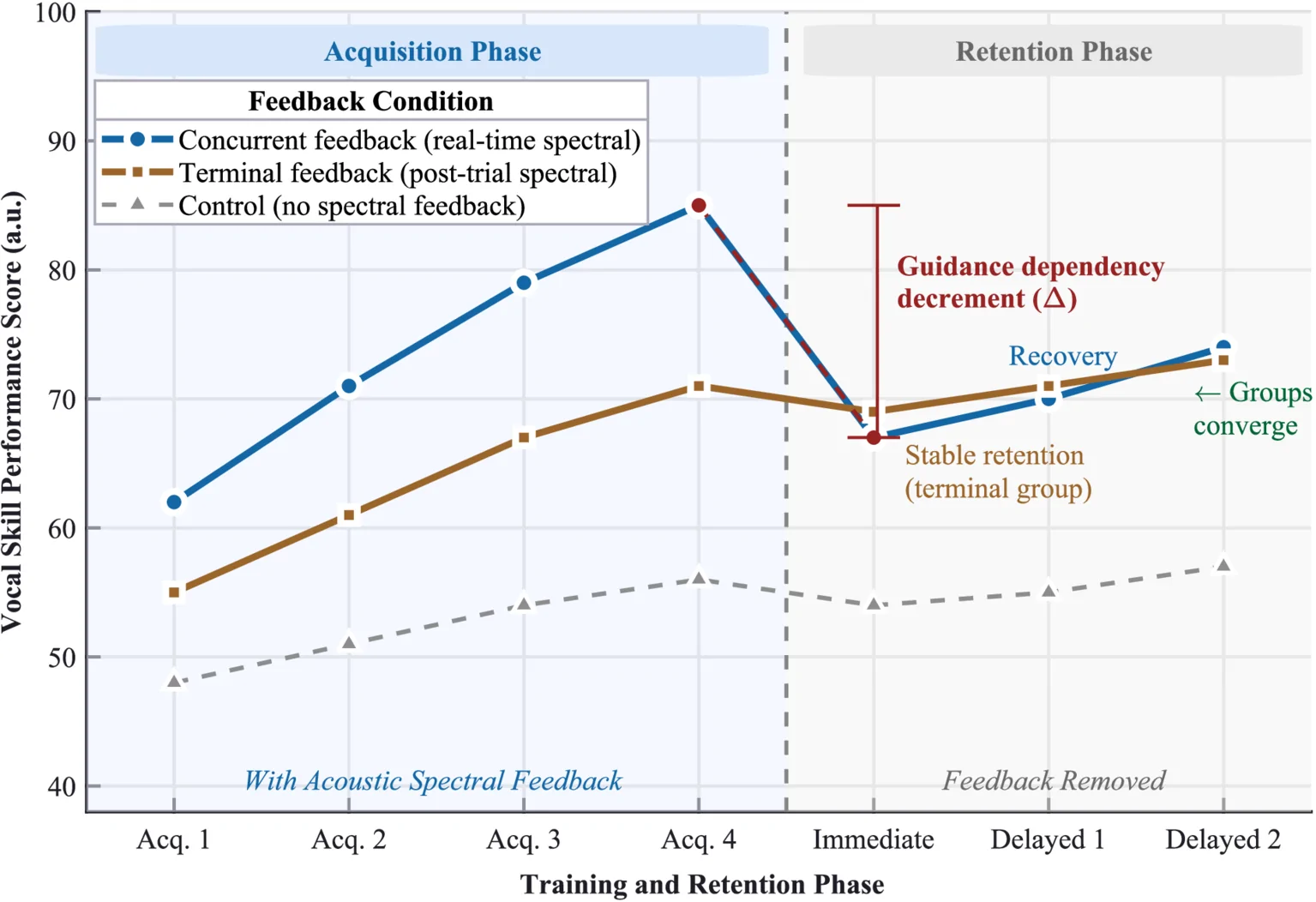
Guidance hypothesis: Concurrent vs. terminal spectral feedback in vocal skill acquisition and retention. Conceptual diagram based on the guidance hypothesis (Salmoni et al., 1984; McKay et al., 2022) adapted to acoustic spectral feedback in vocal learning (Paney and Tharp, 2021; Berglin et al., 2022). Y-axis values are illustrative (arbitrary units, a.u.) and do not represent empirical data. Concurrent feedback tends to elevate acquisition-phase performance but risks guidance dependency upon removal; terminal feedback produces more stable long-term retention. Both groups show convergence at delayed retention, consistent with internalization of the sensorimotor model.

Cognitive load theory (Sweller, 2020) provides a complementary framework for understanding the potential negative effects of concurrent feedback. While supporting error monitoring, a real-time spectral display also introduces additional extraneous cognitive load. Learners need to process both the phonatory action itself and the constantly changing visual information on the screen. For beginners with limited working-memory capacity, this may constitute cognitive overload, which causes performance during training to fall below the no-feedback condition (Jimenez-Diaz et al., 2021; Oppici et al., 2021). Figure 4 illustrates, at the conceptual level, the differential effect curves of concurrent and terminal feedback in the training phase and the retention phase.

The separation of the concurrent and terminal feedback effect curves revealed in Figure 4 has a direct methodological implication for vocal teaching practice. In the beginner phase, concurrent feedback (especially a simplified pitch-trajectory display) can be used to guide the establishment of the sensorimotor mapping. In the skill-consolidation phase, instruction can gradually transition to terminal feedback to promote the internalization of autonomous monitoring ability. This phased strategy holds promise for maximizing the benefit of the tools while controlling the risk of guidance dependency. However, to date, no long-term longitudinal study of vocal learners has systematically tested the timing of this transition (McKay et al., 2022; Drews et al., 2024).

### 3.8 Skill outcomes: a review of measurement tools across dimensions

The vocal skill outcomes reported in the included studies are concentrated in four dimensions. The first is pitch accuracy, measured with automated tools such as the SSAP. Its advantages are that the measurement is objective and repeatable, but it covers only the single dimension of pitch. The second is resonance and timbre, mostly reported through expert auditory-perceptual ratings or spectral feature indices, for which inter-rater reliability varies considerably. The third is breath management, dominated by physiological indices such as subglottal pressure and airflow, and appearing mainly in laboratory settings. The fourth is overall singing performance, represented by multidimensional perceptual rating tools such as the APRIOSV scale, oriented toward the professional assessment of bel canto singing (Oates et al., 2006; Merrill, 2023; Pfordresher and Greenspon, 2025). It is worth noting that the four skill dimensions correspond to different types of spectral feedback. Pitch-accuracy training relies mainly on KR-concurrent tools, whereas resonance/timbre training—which is of more core value for professional vocal pedagogy—relies on KP-type tools (spectrograms and formant displays). Among the included studies, the latter were generally of lower experimental design quality, and the degree of standardization of their measurement tools also urgently needs improvement (Berglin et al., 2022; Moss, 2021).

### 3.9 Current status of mechanism→skill pathway testing

This review conducted a dedicated statistical examination of the 36 included studies. Only 7 studies (19%) explicitly reported a formal mediation pathway test (such as SEM, path analysis, or a mediation–moderation model). The remaining 29 studies (81%) either presented only the independent effects of psychological and skill outcomes (descriptive reporting) or inferred a connection between the two through verbal reasoning (indirect inference), without providing pathway evidence in a statistical sense. As shown in Table 2, among the five psychological mechanisms, the self-efficacy→skill pathway received the most mediation tests (3 studies), all of which were conducted in instrumental rather than vocal settings, the metacognition→skill and SRL→skill pathways had 2 studies each, and the motivation→skill (0 studies) and cognitive-load→skill (1 study) pathways had almost no direct pathway-testing evidence (1 study tested two pathways within the same model, so the path count is 8 while the study count is 7).

**Table 2:** Summary of the current status of mechanism→skill pathway testing.

Psychological mechanism	Total included studies	Formal mediation tests	Direction of conclusion	Representative studies	Path status
Self-efficacy	18	3	Positive (MLSE/MPSE → skill)	Ou et al. (2025) and Zelenak (2024)	Partially tested
Metacognition	12	2	Non-significant (Li et al., 2025); positive (indirect inference)	Li et al. (2025) and Wang et al. (2025)	Path evidence uncertain
SRL cycle	14	2	Positive (mainly qualitative evidence)	López-Íñiguez and McPherson (2020)	Mainly indirect inference
Motivation/emotion	8	0	Indirect inference (descriptive)	Peistaraite and Clark (2020)	Path not tested
Cognitive load/guidance dependency	11	1	Mixed (dependency vs. internalization)	Paney and Tharp (2021) and McKay et al. (2022)	Indirect inference

A formal mediation test refers to a study that uses structural equation modeling, path analysis, or a linear mixed-effects model to statistically test the feedback→mechanism→skill pathway. Indirect inference refers to a study that reports the separate effects of psychological and skill outcomes and infers a connection between them in its discussion, but does not provide a formal pathway statistic. “Path not tested” refers to the absence of both direct measurement and statistical inference. The formal mediation tests recorded at the self-efficacy node were conducted in instrumental settings and are retained as peripheral evidence, whereas the only formal test conducted in a vocal setting returned a non-significant estimate (Li et al., 2025).

The distribution of evidence presented in Table 2 reflects a systematic skew in the research design of this field. The vast majority of studies treat spectral feedback as the independent variable and treat psychological or skill variables as dependent variables, and the design’s endpoint is effect testing rather than mechanism revelation. This pattern is highly consistent with the synthesized conclusion of Wang et al. (2025) on SRL intervention research in music education, namely that single-mechanism interventions often produce local or proximal improvement but rarely reach the learner’s more macro-level SRL and metacognitive patterns. When the pathway-testing gap exists so systematically, existing research has in fact not yet provided a sufficient empirical answer to the question of through which psychological pathway spectral feedback improves vocal skill.

## 4 Discussion

### 4.1 Answers to the research questions

The synthesized evidence permits a direct response to the four questions posed in the Introduction, and this response supplies the ground on which the conceptual framework outlined below is built. In answer to RQ1, the affordances of acoustic spectral feedback in vocal learning are describable through the crossing of feedback nature with feedback timing, which yields the four quadrants of the classification matrix presented in Figure 3, and the distribution of studies across those quadrants is markedly uneven, since the KR-concurrent quadrant carries most of the controlled evidence whereas the two KP quadrants that correspond to resonance regulation remain thinly populated. In answer to RQ2, five mechanisms are advanced in the literature as carriers of the feedback effect, and the density of their direct measurement evidence differs sharply, with self-efficacy and metacognition measured in a substantial number of studies, the sfelf-regulated learning cycle documented mainly through qualitative and correlational work, and motivation together with emotional engagement measured by standardized instruments in no more than five of the 36 included studies. In answer to RQ3, the mediation evidence is considerably weaker than the measurement evidence, because only 7 of the 36 included studies reported a formal test of the feedback→mechanism→skill pathway, the single such test conducted in a vocal setting returned a non-significant metacognition→performance link (Li et al., 2025), and the formally tested and positive mechanism→skill estimates available in the corpus originate in instrumental peripheral studies whose transfer to the vocal domain is an assumption rather than a finding (Ou et al., 2025). In answer to RQ4, the gaps that follow are specific rather than general, and they concern the absence of delayed retention testing under manipulated feedback timing, the absence of longitudinal designs that measure psychological mechanism and vocal skill within the same participants at multiple time points, and the absence of studies recruiting bel canto learners for the formant-related outcomes that KP tools are best positioned to address, which are precisely the gaps that the three hypotheses in Section 4.7 are formulated to close.

### 4.2 Preliminary conceptual framework linking affordance, mechanism, and skill

The evidence synthesized across the five mechanism pathways supports an interpretive proposition rather than a verified mechanism, and it is in this restricted sense that a preliminary three-layer conceptual framework is offered (as shown in Figure 2). The framework introduces no finding additional to the synthesis, since every element within it restates evidence already reported in Sections 3.2 to 3.9, and its function is to hold the answers to RQ1, RQ2, and RQ3 within a single representation from which the design gaps identified under RQ4 can be read directly. What the framework is intended to explain is therefore the evidential architecture of the field rather than the causal structure of vocal learning, in that it makes visible which links between affordance, psychological mechanism, and skill outcome rest on formal tests, which rest on indirect inference, and which have not been examined at all, and it carries no claim that the depicted pathways have been causally verified. The left layer of the framework contains four types of affordance tools, distributed along the two dimensions of KR/KP and concurrent/terminal. The middle layer contains five psychological mechanisms, distinguished by evidence-strength badges (STRONG/MODERATE/LIMITED) that separate direct evidence from inferred pathways. The right layer contains four types of skill outcome. A solid arrow indicates that at least one study formally and statistically tested the pathway and found a result that was significant and positive. A pathway that was formally tested but found non-significant (such as metacognition→singing performance) is marked separately and is not counted as a solid arrow. A dashed arrow indicates a hypothesized pathway that relies on indirect inference. A question mark marks a connection for which no direct pathway test yet exists (such as motivation→overall singing performance).

Figure 2 reveals several deeper features of the current evidence landscape in this field. Between the middle layer and the right layer of the framework, no mechanism→skill link examined in a vocal setting has been formally tested and significantly supported, the single formally tested link in that setting returned a non-significant result (metacognition→singing performance), and the positive mediation estimates recorded for the self-efficacy node in Table 2 derive from instrumental peripheral studies rather than from vocal ones, with the consequence that every remaining mechanism→skill link rests on indirect inference or has not been tested at all. This imbalance in proportions is not accidental but reflects a structural shortcoming, namely that research design in this field systematically stops at effect testing and fails to advance to mechanism verification. More noteworthy is that the pathways between the motivation/emotional-engagement mechanism (M4) and all skill outcomes are all marked with question marks. This means that, in the field of vocal spectral feedback, the theoretical status of motivation as a mediating variable in the learning process has not yet received any direct statistical-test support, and it constitutes the weakest evidence node in the framework (Wang et al., 2025; Ou et al., 2025).

### 4.3 Complementary integration of the motor learning and SRL frameworks

The core theoretical contribution of the conceptual framework advanced in this review lies in positioning the motor learning framework and the SRL/metacognition framework as complementary rather than competing levels of explanation. Motor learning theory (especially the guidance hypothesis and cognitive load theory) mainly explains how external feedback is utilized by the sensorimotor system. The spectral signal provides the precise error information that internal perception lacks, and it activates the corrective process of the sensorimotor mapping (McKay et al., 2022; Sweller, 2020). The SRL/metacognition framework, by contrast, mainly explains how internal cognitive–emotional regulation mediates the long-term influence of this external feedback on skill development. Objective information from a tool is not in itself sufficient to produce a lasting improvement in skill. Learners also need to integrate this information into autonomous goal setting, strategy selection, and the self-reflection cycle (López-Íñiguez and McPherson, 2020; Hatfield et al., 2017; Panadero and Lipnevich, 2022). The point at which the two frameworks meet is this: as an external trigger, spectral feedback lowers the cognitive cost of monitoring, thereby providing higher-quality information input for each phase of the SRL cycle and, in turn, strengthening the construction of the internal sensorimotor model (Ritchie and Williamon, 2011; Peistaraite and Clark, 2020).

### 4.4 Relationship to and increment over existing reviews

With respect to its relationship to existing reviews, the positioning and contribution of this study need to be defined carefully. The PRISMA review by Blackwell et al. (2023) systematically mapped the overall landscape of feedback research in music education and music psychology, but its unit of analysis was the feedback type rather than the psychological mechanism, and it did not focus on vocal spectral visualization tools. The systematic review and meta-analysis by Wang et al. (2025) on metacognition and SRL intervention research in music education is the closest to this review in research orientation, but it covered music education in the broad sense (including instrumental music, choir, music theory, and the like), did not treat vocal spectral feedback as an independent analytical category, and did not distinguish the mechanism differences along the KR/KP or concurrent/terminal dimensions. The review by Lubert and Gröpel (2025) focused on self-regulated enhancement interventions for music performance; it likewise addressed broad instrumental/vocal settings, and its analytical framework was based on intervention type rather than affordance classification. Compared with these three reviews, the increment of this study is reflected specifically in the following. It proposes an affordance classification matrix specific to vocal spectral feedback (innovation point ①). It systematically annotates the differential evidence strength of the five mechanism pathways (innovation point ②). It quantifies the proportion of “untested pathways” and presents the evidence gaps in tabular form (innovation point ③). It advances a three-layer conceptual framework whose links are stated as testable propositions for subsequent research rather than as verified mechanisms (innovation point ④). It should be emphasized that all of the contributions above rest on the epistemological premise of a “preliminary synthesis,” and the causal pathways depicted in the framework await verification by more rigorous experimental designs (Wang et al., 2025; Blackwell et al., 2023; Lubert and Gröpel, 2025).

### 4.5 Practical implications for vocal pedagogy

The existing evidence offers several limited but valuable methodological references for vocal teaching practice. For beginners, KR-concurrent tools (such as a simplified pitch-trajectory display) have relatively good evidential support for helping to establish the basic pitch-perception–phonation coordination mapping, and teachers can use them as a scaffold during the error-identification phase. In view of the risk of guidance dependency, however, we recommend a gradual transition to terminal feedback once the learner has reached a basic level of accuracy (Bernardini et al., 2022; Paney and Tharp, 2021). For professional bel canto pedagogy, formant and broadband spectral displays have a theoretical advantage in externalizing the regulation of vocal-tract resonance, but the controlled experimental evidence supporting this application scenario is extremely limited; teachers should position such tools as auxiliary means of observation rather than as standalone instructional interventions (Moss, 2021; Oppici et al., 2021). More importantly, a consistent finding from SRL research indicates that the way a tool is embedded influences the learning outcome no less than the tool itself. Using a spectral display tool in isolation, without integrating it into an explicit cycle of goal setting, monitoring, and reflection, will greatly reduce its benefit (Wan et al., 2023; McPherson et al., 2022; Suzuki et al., 2024). It should be emphasized again that the existing evidence comes mostly from general singing-learning settings. Spectral feedback research dedicated to bel canto training—especially formant regulation and passaggio control—is extremely scarce, and the over-extrapolation of clinical teaching recommendations lacks sufficient support at the level of evidence (Moss, 2021).

### 4.6 Limitations

This review has several limitations that must be honestly acknowledged. First, the high heterogeneity of the included studies makes cross-study comparison difficult at the level of many details. The study participants span a broad range from complete beginners to professional singers, the feedback tools used differ greatly in functional complexity and mode of presentation, and the degree of standardization of the measurement tools is uneven. These factors together make it difficult for this review to provide a precise quantitative synthesis conclusion of the kind that a meta-analysis would offer. The choice of narrative synthesis is therefore a methodological necessity rather than a matter of convenience (Wang et al., 2025). Second, although this review included some Chinese-language literature through the CNKI search, English-language literature still predominates overall, and vocal spectral feedback research published in Chinese journals may be systematically underestimated. This is especially regrettable for understanding the specific evidence on bel canto teaching practice in Chinese higher-education institutions. Third, among the 36 included studies, only 22 simultaneously satisfied the three elements of “spectral feedback + psychological mechanism measurement + vocal learning context,” and this relatively small core evidence base limits the depth of the mediation mechanism analysis. Fourth, owing to the existence of publication bias, studies with positive effects systematically enter the literature pool more readily than null results, so this review’s synthesized assessment of the benefit of spectral feedback may involve a degree of overestimation (Page et al., 2021; Wang et al., 2025). A further limitation concerns the analytic process itself. The screening, charting, and mechanism assignment described in Section 2.8 were carried out by a single author, and the procedural safeguards adopted there, namely an *a priori* coding frame, duplicated passes separated in time, an explicit search for disconfirming evidence, and a retained audit trail, constrain but cannot fully substitute for the independent inter-coder checking and external auditing that a collaborative team is able to perform. The judgments most exposed to this constraint are the assignment of borderline studies to mechanism nodes and the evidence-strength annotation displayed in Figure 2, both of which involve interpretive decisions that another analyst might reasonably resolve differently, and the synthesized conclusions should therefore be weighed with that residual subjectivity in view.

### 4.7 Research agenda

Based on the evidence gaps above, this review proposes three operationalizable and testable research hypotheses, with the aim of providing clear directional guidance for subsequent empirical research. Hypothesis 1: Compared with terminal feedback, concurrent feedback produces a higher immediate performance level during the acquisition phase of vocal skill, but this advantage disappears or even reverses in a delayed retention test. This hypothesis needs to be tested through a randomized controlled trial that includes a delayed retention test, with feedback timing (concurrent vs. terminal) as the independently manipulated variable (Paney and Tharp, 2021; McKay et al., 2022). Hypothesis 2: Spectral feedback influences vocal skill acquisition through the mediation chain of objective improvement in pitch/resonance accuracy→enhanced self-efficacy→sustained practice behavior→long-term skill development. Testing this pathway requires a longitudinal design with multiple time points that measures self-efficacy (MLSE/MPSE scales), practice behavior (practice logs), and skill outcomes within the same study, and estimates the mediation effect through structural equation modeling (Ritchie and Williamon, 2011; Zelenak, 2024; Ou et al., 2025). Hypothesis 3: The facilitating effect of spectral feedback in vocal training on formant-related skills (such as the development of the singer’s formant and the vocal-tract regulation of the passaggio region) is specific to bel canto and cannot be replaced by evidence drawn from general singing learners. Testing this hypothesis requires specifically recruiting bel canto vocal students and using a dual outcome design of acoustic-parameter measurement (F1–F2 trajectory, spectral tilt, and the like) and expert rating (Zelenak, 2024).

## 5 Conclusion

This review synthesizes the role of acoustic spectral feedback in vocal learning with psychological mechanisms as its explicit organizing framework, an axis that previous reviews of feedback in music learning have not taken as their unit of analysis. By integrating motor learning theory and the self-regulated learning framework, this review identified five psychological mechanism pathways with differentiated evidence strength, advanced a preliminary conceptual framework whose pathways are offered as testable propositions rather than as established mechanisms, and systematically annotated the most prominent methodological gap in the current field, namely that the vast majority of studies stop at effect testing and fail to advance to the verification of mediation pathways. Together, these findings point to a less optimistic but more realistic evidence picture: the psychological mechanisms of spectral feedback in vocal learning still remain, to a considerable extent, at the level of theoretical hypothesis and indirect inference, rather than being causal propositions tested by rigorous experimental design. This situation is both a limitation and an opportunity. For researchers who wish to advance theoretical construction in this field, carefully designed mediation tests, multi-time-point longitudinal tracking, and dedicated empirical research targeting bel canto-specific settings will constitute the most academically valuable research directions.

## Data Availability

The original contributions presented in the study are included in the article/Supplementary material, further inquiries can be directed to the corresponding author.
