# Supplementary material for: Acoustic spectral feedback in vocal learning: a systematic review of psychological mechanisms and skill outcomes
Source: Front Psychol. 2026 Aug 20;17:1920074. doi: 10.3389/fpsyg.2026.1920074 (PMC13537971; doi:10.3389/fpsyg.2026.1920074)
Supplement: Supplementary file 1 [file Supplementary_file_1.DOCX]

**PRISMA 2020 Checklist**

*Supplementary Material for: “Acoustic Spectral Feedback in Vocal Learning: A Systematic Review of Psychological Mechanisms and Skill Outcomes”*

| **Section and Topic** | **Item #** | **Checklist item** | **Location where item is reported** |
| --- | --- | --- | --- |
| **TITLE** | | | |
| **Title** | **1** | Identify the report as a systematic review. | Title page (title states “A Systematic Review”). |
| **ABSTRACT** | | | |
| **Abstract** | **2** | See the PRISMA 2020 for Abstracts checklist. | Abstract (structured: Background, Aim, Methods, Results, Conclusion). |
| **INTRODUCTION** | | | |
| **Rationale** | **3** | Describe the rationale for the review in the context of existing knowledge. | Section 1 (Introduction), paras 1–4. |
| **Objectives** | **4** | Provide an explicit statement of the objective(s) or question(s) the review addresses. | Section 1 (Introduction), final para (four research questions). |
| **METHODS** | | | |
| **Eligibility criteria** | **5** | Specify the inclusion and exclusion criteria for the review and how studies were grouped for the syntheses. | Section 2.3 (Inclusion and Exclusion Criteria); grouping in Section 2.7. |
| **Information sources** | **6** | Specify all databases, registers, websites, organisations, reference lists and other sources searched or consulted to identify studies. Specify the date when each source was last searched or consulted. | Section 2.2 (WoS, Scopus, APA PsycINFO, ERIC, PubMed, CNKI; searched through August 2025). |
| **Search strategy** | **7** | Present the full search strategies for all databases, registers and websites, including any filters and limits used. | Section 2.2 (core Boolean string and PCC framework). Full per-database strings: Supplementary Material. |
| **Selection process** | **8** | Specify the methods used to decide whether a study met the inclusion criteria of the review, including how many reviewers screened each record and each report retrieved, whether they worked independently, and if applicable, details of automation tools used in the process. | Section 2.4 (single author; two-stage screening, performed twice). No automation tools used. |
| **Data collection process** | **9** | Specify the methods used to collect data from reports, including how many reviewers collected data from each report, whether they worked independently, any processes for obtaining or confirming data from study investigators, and if applicable, details of automation tools used in the process. | Section 2.5 (predefined charting form; single author, double-checked). |
| **Data items** | **10a** | List and define all outcomes for which data were sought. Specify whether all results that were compatible with each outcome domain in each study were sought (e.g. for all measures, time points, analyses), and if not, the methods used to decide which results to collect. | Section 2.5 (psychological outcome and skill-outcome fields); outcome domains detailed in Section 3.8. |
|  | **10b** | List and define all other variables for which data were sought (e.g. participant and intervention characteristics, funding sources). Describe any assumptions made about any missing or unclear information. | Section 2.5 (sample N/type, feedback type, timing, KR/KP, mediation-test status). |
| **Study risk of bias assessment** | **11** | Specify the methods used to assess risk of bias in the included studies, including details of the tool(s) used, how many reviewers assessed each study and whether they worked independently, and if applicable, details of automation tools used in the process. | Section 2.6 (Mixed Methods Appraisal Tool, MMAT). Per-study ratings: recommended for Supplementary Material. |
| **Effect measures** | **12** | Specify for each outcome the effect measure(s) (e.g. risk ratio, mean difference) used in the synthesis or presentation of results. | Section 2.7 / Section 3 — direction-of-effect synthesis; effect sizes reported by individual studies are presented narratively (e.g. d, η²p). No pooled effect measure (no meta-analysis). |
| **Synthesis methods** | **13a** | Describe the processes used to decide which studies were eligible for each synthesis (e.g. tabulating the study intervention characteristics and comparing against the planned groups for each synthesis (item #5)). | Section 2.7 (synthesis unit = psychological mechanism); Section 3.1; Table 1. |
|  | **13b** | Describe any methods required to prepare the data for presentation or synthesis, such as handling of missing summary statistics, or data conversions. | Section 2.5 / 2.7. No statistical data conversion performed (narrative synthesis). |
|  | **13c** | Describe any methods used to tabulate or visually display results of individual studies and syntheses. | Section 2.7; Tables 1–2; Figures 2–4. |
|  | **13d** | Describe any methods used to synthesize results and provide a rationale for the choice(s). If meta-analysis was performed, describe the model(s), method(s) to identify the presence and extent of statistical heterogeneity, and software package(s) used. | Section 2.1 and 2.7 (thematic narrative synthesis; explicit rationale given for not performing a meta-analysis). |
|  | **13e** | Describe any methods used to explore possible causes of heterogeneity among study results (e.g. subgroup analysis, meta-regression). | Section 2.7 — heterogeneity explored narratively across mechanism nodes (e.g. KR/KP, timing, learner level). Statistical heterogeneity: Not applicable — narrative/thematic synthesis (no meta-analysis); see Section 2.1. |
|  | **13f** | Describe any sensitivity analyses conducted to assess robustness of the synthesized results. | Not applicable — narrative/thematic synthesis (no meta-analysis); see Section 2.1. |
| **Reporting bias assessment** | **14** | Describe any methods used to assess risk of bias due to missing results in a synthesis (arising from reporting biases). | Addressed narratively; publication bias discussed in Section 4.5. Funnel-plot/regression tests not applicable (no meta-analysis). |
| **Certainty assessment** | **15** | Describe any methods used to assess certainty (or confidence) in the body of evidence for an outcome. | Section 2.6 — MMAT plus evidence-strength badges (STRONG / MODERATE / LIMITED) defined by number of studies directly measuring each mechanism. Formal GRADE not used. |
| **RESULTS** | | | |
| **Study selection** | **16a** | Describe the results of the search and selection process, from the number of records identified in the search to the number of studies included in the review, ideally using a flow diagram. | Section 2.4 and Section 3.1; Figure 1 (PRISMA 2020 flow diagram). |
|  | **16b** | Cite studies that might appear to meet the inclusion criteria, but which were excluded, and explain why they were excluded. | Figure 1 (exclusion counts by stage). A list of full-text exclusions with reasons is recommended for the Supplementary Material. |
| **Study characteristics** | **17** | Cite each included study and present its characteristics. | Section 3.1; Table 1 (representative studies). Full characteristics table: Supplementary Material. |
| **Risk of bias in studies** | **18** | Present assessments of risk of bias for each included study. | Section 4.5 and discussion of MMAT ratings (Section 2.6). Per-study RoB table recommended for Supplementary Material. |
| **Results of individual studies** | **19** | For all outcomes, present, for each study: (a) summary statistics for each group (where appropriate) and (b) an effect estimate and its precision (e.g. confidence/credible interval), ideally using structured tables or plots. | Table 1; Sections 3.3–3.9 (individual-study results presented narratively, including reported statistics such as d, η²p, F, t). |
| **Results of syntheses** | **20a** | For each synthesis, briefly summarise the characteristics and risk of bias among contributing studies. | Sections 3.2–3.9 (per-mechanism syntheses). |
|  | **20b** | Present results of all statistical syntheses conducted. If meta-analysis was done, present for each the summary estimate and its precision (e.g. confidence/credible interval) and measures of statistical heterogeneity. If comparing groups, describe the direction of the effect. | Sections 3.3–3.9 — direction-of-effect narrative synthesis by mechanism; Section 3.9 and Table 2 (pathway-testing status). No pooled statistical synthesis (no meta-analysis). |
|  | **20c** | Present results of all investigations of possible causes of heterogeneity among study results. | Sections 3.2–3.9 and Table 2 (heterogeneity discussed narratively, e.g. moderation by initial ability level; KR vs. KP). |
|  | **20d** | Present results of all sensitivity analyses conducted to assess the robustness of the synthesized results. | Not applicable — narrative/thematic synthesis (no meta-analysis); see Section 2.1. |
| **Reporting biases** | **21** | Present assessments of risk of bias due to missing results (arising from reporting biases) for each synthesis assessed. | Section 4.5 (publication bias discussed narratively). |
| **Certainty of evidence** | **22** | Present assessments of certainty (or confidence) in the body of evidence for each outcome assessed. | Sections 3.3–3.9; Figure 4 (evidence-strength badges); Table 2. |
| **DISCUSSION** | | | |
| **Discussion** | **23a** | Provide a general interpretation of the results in the context of other evidence. | Sections 4.1–4.3. |
|  | **23b** | Discuss any limitations of the evidence included in the review. | Section 4.5 (Limitations). |
|  | **23c** | Discuss any limitations of the review processes used. | Section 4.5 (Limitations). |
|  | **23d** | Discuss implications of the results for practice, policy, and future research. | Sections 4.4 and 4.6; Section 5 (Conclusion). |
| **OTHER INFORMATION** | | | |
| **Registration and protocol** | **24a** | Provide registration information for the review, including register name and registration number, or state that the review was not registered. | Section 2.1 (pre-registered on the Open Science Framework, OSF). Add the OSF registration ID/DOI here before submission. |
|  | **24b** | Indicate where the review protocol can be accessed, or state that a protocol was not prepared. | Section 2.1 (protocol pre-registered on OSF). Add the accessible protocol link before submission. |
|  | **24c** | Describe and explain any amendments to information provided at registration or in the protocol. | Section 2.1 (states that any deviations from the pre-registered protocol are noted in the Methods). |
| **Support** | **25** | Describe sources of financial or non-financial support for the review, and the role of the funders or sponsors in the review. | Funding section. Complete with funder details/grant numbers (or a “no funding” statement) before submission. |
| **Competing interests** | **26** | Declare any competing interests of review authors. | Conflict of Interest statement. Complete before submission. |
| **Availability of data, code and other materials** | **27** | Report which of the following are publicly available and where they can be found: template data collection forms; data extracted from included studies; data used for all analyses; analytic code; any other materials used in the review. | Data Availability Statement. This Supplementary Material provides the PRISMA 2020 checklist, the search strategy, and the data-extraction (charting) form. |

*From: Page MJ, McKenzie JE, Bossuyt PM, et al. The PRISMA 2020 statement: an updated guideline for reporting systematic reviews. PLOS Medicine 2021;18(3):e1003583. doi:10.1371/journal.pmed.1003583*

*For more information, visit: http://www.prisma-statement.org/*
